# Measuring Resilience and Resistance in Aging and Alzheimer Disease Using Residual Methods: A Systematic Review and Meta-analysis

**DOI:** 10.1212/WNL.0000000000012499

**Published:** 2021-09-07

**Authors:** Diana I. Bocancea, Anna C. van Loenhoud, Colin Groot, Frederik Barkhof, Wiesje M. van der Flier, Rik Ossenkoppele

**Affiliations:** From the Alzheimer Center Amsterdam, Department of Neurology, Amsterdam Neuroscience (D.I.B., A.C.v.L., C.G., W.M.v.d.F., R.O.), and Department of Radiology and Nuclear Medicine (F.B.), Vrije Universiteit Amsterdam, Amsterdam UMC, the Netherlands; Institutes of Neurology and Healthcare Engineering (F.B.), University College London, UK; Department of Epidemiology and Biostatistics (W.M.v.d.F.), VU University Medical Center, Amsterdam, the Netherlands; and Clinical Memory Research Unit (R.O.), Lund University, Sweden.

## Abstract

**Background and Objective:**

There is a lack of consensus on how to optimally define and measure resistance and resilience in brain and cognitive aging. Residual methods use residuals from regression analysis to quantify the capacity to avoid (resistance) or cope (resilience) “better or worse than expected” given a certain level of risk or cerebral damage. We reviewed the rapidly growing literature on residual methods in the context of aging and Alzheimer disease (AD) and performed meta-analyses to investigate associations of residual method–based resilience and resistance measures with longitudinal cognitive and clinical outcomes.

**Methods:**

A systematic literature search of PubMed and Web of Science databases (consulted until March 2020) and subsequent screening led to 54 studies fulfilling eligibility criteria, including 10 studies suitable for the meta-analyses.

**Results:**

We identified articles using residual methods aimed at quantifying resistance (n = 33), cognitive resilience (n = 23), and brain resilience (n = 2). Critical examination of the literature revealed that there is considerable methodologic variability in how the residual measures were derived and validated. Despite methodologic differences across studies, meta-analytic assessments showed significant associations of levels of resistance (hazard ratio [HR] [95% confidence interval (CI)] 1.12 [1.07–1.17]; *p* < 0.0001) and levels of resilience (HR [95% CI] 0.46 [0.32–0.68]; *p* < 0.001) with risk of progression to dementia/AD. Resilience was also associated with rate of cognitive decline (β [95% CI] 0.05 [0.01–0.08]; *p* < 0.01).

**Discussion:**

This review and meta-analysis supports the usefulness of residual methods as appropriate measures of resilience and resistance, as they capture clinically meaningful information in aging and AD. More rigorous methodologic standardization is needed to increase comparability across studies and, ultimately, application in clinical practice.

Heterogeneity in brain and cognitive aging has been observed in numerous studies, with some individuals aging more successfully than others. Some people are less susceptible to age-associated or neurodegenerative pathologic changes, while others preserve brain integrity and cognitive abilities despite emerging neuropathology. Various heuristic constructs (e.g., cognitive/brain reserve, neural compensation, brain maintenance) have been proposed to describe different aspects of the observed individual differences in brain and cognitive aging trajectories.^1,2^ Here, we adopt and expand upon a previously proposed framework^3,4^ that conceptualizes 2 distinct phenomena under the umbrella terms resistance and resilience. Resistance is defined as the brain's ability to avoid age-related senescent and pathologic changes, thereby preserving brain integrity and cognition, despite risk factors such as advanced age or genetic predisposition to Alzheimer disease (AD) pathology. Resilience refers to the brain's ability to cope with accumulating senescent and pathologic changes and preserve brain integrity (brain resilience) or cognitive function (cognitive resilience) in the face of significant pathologic burden.^3,4^ For example, an elderly *APOE*ε4/ε4 carrier is at increased risk of accumulating AD neuropathology and subsequent cognitive impairment. Remaining relatively free of neuropathology would indicate resistance, while preserving normal cognition despite β-amyloid and tau accumulation indicates (cognitive) resilience. Furthermore, cognitive preservation could be explained by maintenance of structural integrity despite pathology, indicating brain resilience. The conceptual model in Figure 1 characterizes an individual's level of cognitive aging as a combination of resistance and resilience to senescent and pathologic changes. These 2 modes of cognitive preservation are distinct, yet not mutually exclusive, determinants of successful cognitive aging.

**Figure 1:**
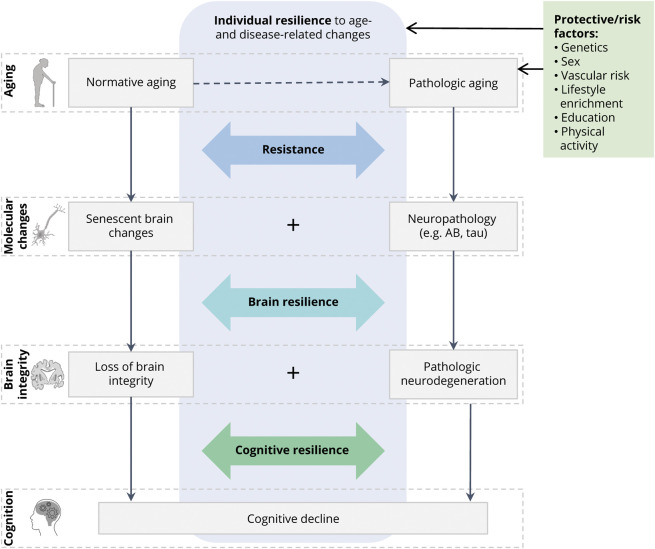
Conceptual Model of Resistance and Resilience in Normative and Pathologic Brain and Cognitive Aging An individual's level of (successful) cognitive aging is determined by 2 distinct modes of cognitive preservation, resistance, and (brain and cognitive) resilience. Recent research in the cognitive decline and dementia field posits neurodegenerative disorders as the product of multiple proteinopathies and other pathologic events occurring in conjunction. Similarly, heterogeneity in cognitive aging trajectories is determined by varying degrees of resistance- and resilience-related mechanisms that interact with these processes and synergistically contribute to successful aging. Icons in this figure are modified from Servier Medical Art, licensed under a Creative Commons Attribution 3.0 Unported License (smart.servier.com/).

## Measuring Resistance and Resilience

Resistance and resilience in the aging brain exist by virtue of both genetics and environmental exposures across the lifespan; for example, education, intellectually engaging leisure activities, or physical exercise.^5,6^ There is no consensus on how to optimally define and measure these constructs. Sociodemographic variables^7,8^ and questionnaire-derived metrics^9^ are commonly used as proxy measures of cognitive reserve, a related construct representing a potential mechanism of resilience. These measures, however, explain only part of the observed variance in successful aging and hamper the study of determinants and mechanisms of resilience and resistance. More recently, statistical methods using residuals from regression analyses (henceforth referred to as “residual methods”) have been developed as alternative operational measures. These methods are based on the observed imbalance between risk factors, age-related neurobiological changes, and clinical expression and therefore enable the computation of individualized measures of resistance and resilience in a more objective manner.

### The Residual Approach

The residual approach operationalizes resistance and resilience as discrepancies between risk factors, cognition, and brain status. Resistance is generally determined by the mismatch between risk factors and brain integrity, while resilience is determined by the mismatch between senescent/neuropathologic changes and cognition (cognitive resilience) or brain structure (brain resilience).

### How Are Residuals Calculated?

**Figure 2:**
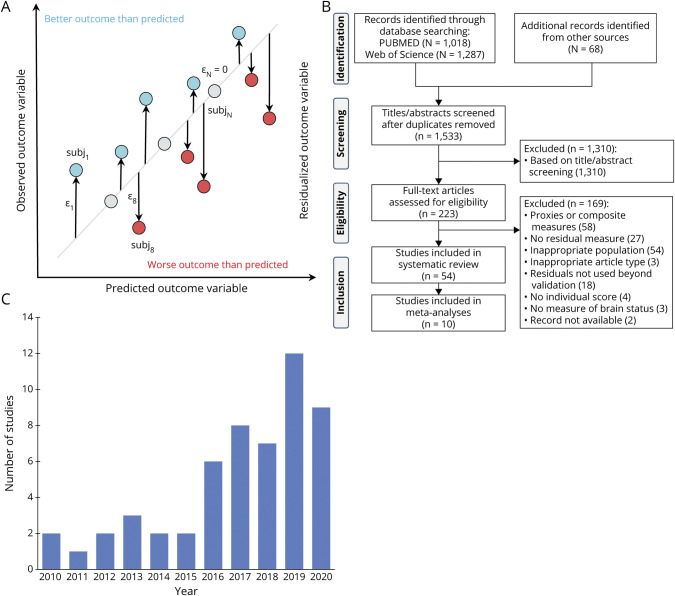
Generic Diagram of the Residual Approach, Study Selection Flowchart, and Histogram of Selected Studies Publication Year (A) Person-specific residuals (ε) are computed as the difference between an observed outcome variable and that predicted by a number of variables of interest. Individuals who present a better outcome than predicted (generally) have a higher level of resilience or resistance. Note however that the directionality of the residuals ultimately depends on the outcome and predictor variables in the model, and hence, in some studies a negative residual indicates higher resilience/resistance. (B) Preferred Reporting Items for Systematic Reviews and Meta-Analyses (PRISMA) flowchart of study selection. (C) Distribution of publication year for the 54 studies eligible for inclusion in the systematic review. This figure illustrates an increasing number of studies over the past decade that use a residual method–based measure of resilience or resistance.

Residual measures result from statistical models that predict the association between variables of brain status (e.g., cerebral volumes or tau accumulation) and participant-specific characteristics (e.g., age or cognition). A residual is calculated as the difference between predicted and observed outcome variables for an individual relative to the population estimate, and therefore, quantifies the extent to which the observed outcome positively or negatively deviates from normative values (Figure 2A). Individuals with better outcome than expected are considered resistant/resilient.

### Aims

Despite decades of study, quantitative assessment of an individual's level of resilience or resistance is not yet implemented in clinical practice and clinical trials. We systematically reviewed the literature on residual measures of resistance- and resilience-related constructs, following the conceptual framework described above. First, we provide a detailed overview of the different residual methods, their methodologic characteristics, analytical approaches, and validation. Second, we investigate how residual measures help explain heterogeneity in brain and cognitive aging by meta-analyzing their associations with cognitive decline and disease progression. Third, we critically appraise the challenges with residual approaches as surrogate measures of resistance and resilience and discuss future perspectives.

## Methods

### Study Selection

This study was conducted following preestablished methods and is reported following Preferred Reporting Items for Systematic Reviews and Meta-Analyses (PRISMA) guidelines^10^ (eTable 1, available from Dryad, doi.org/10.5061/dryad.tx95x69xr). We performed a systematic literature search in PubMed/Medline and Web of Science databases from inception through March 10, 2020. We searched studies that operationalized resistance/resilience-related constructs with a residual method using a combination of relevant terms (see full search queries in eTable 2, available from Dryad, doi.org/10.5061/dryad.tx95x69xr). We included peer-reviewed articles, written in English and presenting original research with human data. Excluding review articles and meta-analyses, all other study designs were eligible. We included studies that (1) operationalized any type of a resistance- or resilience-related construct with residuals, (2) included at least a measure of brain status (i.e., neuroimaging-based brain integrity or brain-derived molecular markers of senescence or pathology) in the model from which residuals were computed, irrespective of where the variables were entered in the regression model, (3) involved samples of individuals across normative aging or AD-related trajectories, (4) derived individual-level residuals, and (5) utilized the residual measure beyond methodologic validation of the model.

Articles were screened at the title/abstract level in Rayyan^11^ (rayyan.qcri.org/). Reference lists were additionally cross-checked for eligible studies. Two authors (D.I.B., A.C.v.L.) reviewed the studies for inclusion and abstracted the data, and ambiguous records/discrepancies were discussed with a third author (R.O.) to reach consensus. For each study, we extracted the sample characteristics, type of residual (i.e., residualized outcome variable), modeled variables and their operationalization, covariates, and analytical methods used for modeling and quantifying the residual measure.

### Meta-analysis

Using meta-analytical approaches, we investigated associations between residual method–based resilience or resistance and cognitive decline or disease progression in individuals across normative aging or AD-related trajectories. We selected studies with appropriate analyses and extracted standardized regression coefficients for cognitive decline and hazard ratios (HRs) for disease progression. Effect estimates were adjusted for multiple variables as they came from models that included varying covariate combinations. HRs and their error estimates were log-transformed for analysis and subsequently transformed back for reporting purposes.^12^ Missing data were requested from the authors of 6 studies^13-18^ (6/6 responded). Due to high heterogeneity in samples, methodology, and outcomes among studies, we hypothesized that the true effect size might be study-dependent. We therefore assessed overall effects using random-effect models^12^ in the R (v3.6.1) metafor package (v2.4-0).^19^ Statistical heterogeneity was assessed using the *I*^*2*^ statistic and Q-test *p* value,^20^ with *I*^*2*^ ≥75% indicating substantial heterogeneity. Significance for random effects was set at *p* < 0.05. Publication bias was assessed by visual inspection of funnel plots.^12^ Two authors (D.I.B., C.G.) independently assessed risk of bias with a domain-based tool.^21^ Furthermore, we evaluated in sensitivity analyses the effect of removing those studies that were rated at a potential risk of bias.

## Results

**Figure 3:**
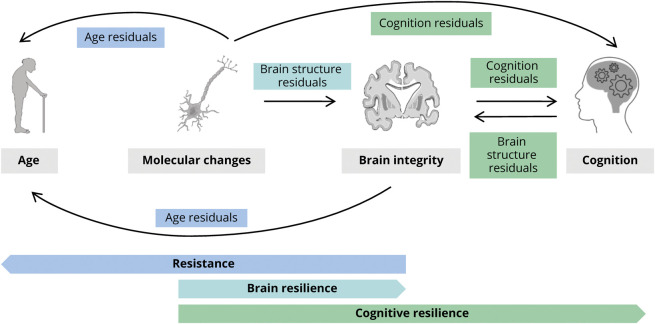
Residuals Measures of Resistance and Resilience-Related Constructs Schematic diagram depicting a gross categorization of the different residual methods we identified in the reviewed studies. Within the framework of resistance and (brain and cognitive) resilience, according to what outcome variable has been modeled and subsequently residualized, we identified age-based, brain-based, and cognition-based residual methods. Arrows point from the predictor variables to the outcome variable residualized (e.g., we found 2 different ways of calculating age-based residuals: predicting chronological age with respect to molecular change measures or with respect to brain integrity measures). Note that while resistance and brain resilience concepts partially overlap, as both include preservation of brain structure (brain integrity), we distinguish them depending on whether risk factor (age) or pathology (molecular changes) is included in the equation. Similarly, brain and cognitive resilience partially overlap, as both may include pathology (molecular changes) as predictors, and we distinguish them based on whether the residualized outcome represents brain integrity or cognition. These 2 models may reflect different phenotypes of resilience, as cognitive resilience relative to the molecular markers could (partly) be explained by brain resilience. Icons in this figure are modified from Servier Medical Art, licensed under a Creative Commons Attribution 3.0 Unported License (smart.servier.com/).

The systematic literature search yielded 1,501 records, with 210 studies assessed at full-text level. Among 54 studies fulfilling inclusion criteria (Figure 2, B and C), we identified 33 resistance-related residuals and 25 resilience-related residuals (Figure 3 and eTable 3, available from Dryad, doi.org/10.5061/dryad.tx95x69xr).

### Residual Measures of Resistance

Resistance-related residuals were typically based on regression models including chronological age and measures of brain integrity, more often with age (age-based residuals) than brain integrity (brain-based residuals) as dependent variable (Table 1 and eTable 5). The specifics of age-based and brain-based residual approaches are outlined below.

**Table 1:**
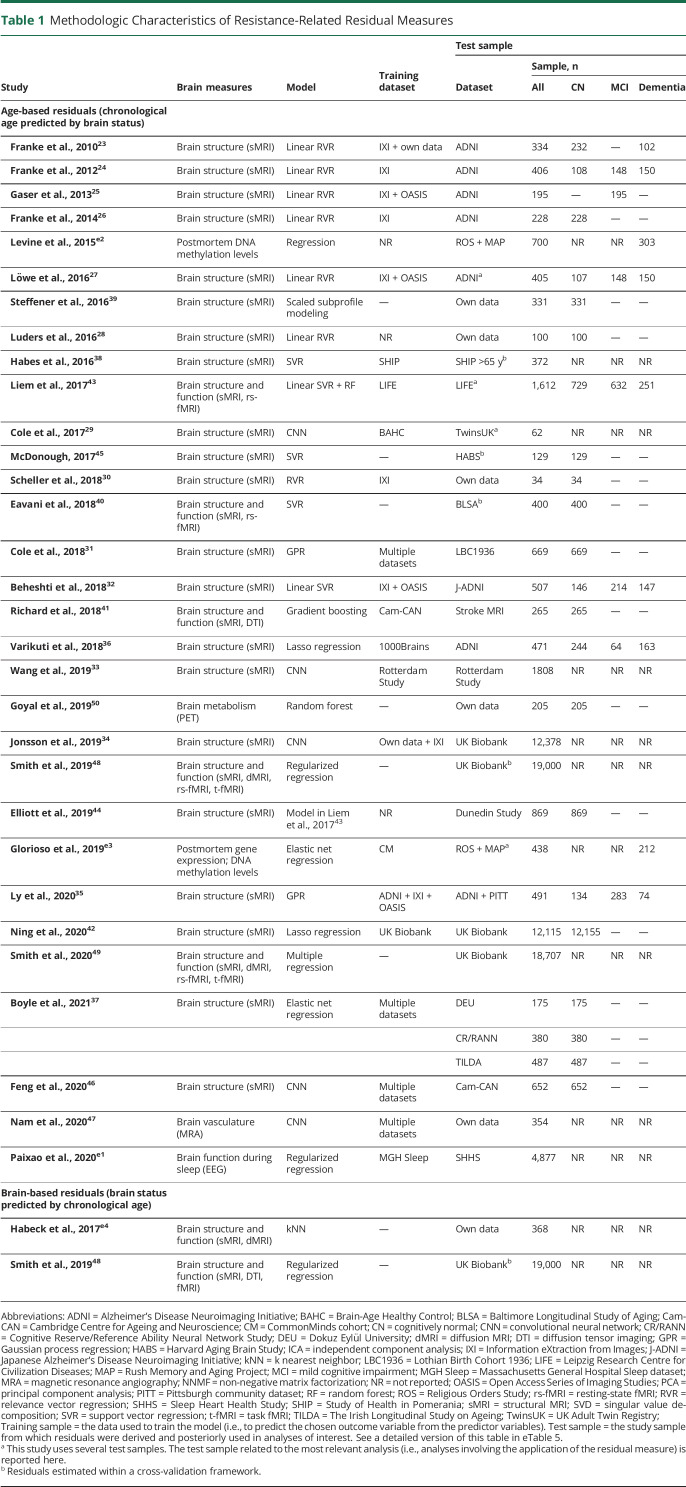
Methodologic Characteristics of Resistance-Related Residual Measures

#### Age-Based Residuals

Age-based residuals (commonly known as “brain age gap” measures) are derived from models that infer the apparent age of an individual from variables reflecting brain status (e.g., whole-brain atrophy) or senescence (e.g., DNA methylation). These models are built to capture the normative aging process in healthy individuals. When subsequently applied to new individuals, residual measures are computed as the difference between the chronological age and predicted brain age. These residuals capture deviation from the population norm, measuring the extent to which patients preserve (structural, functional, or molecular) brain integrity despite chronological aging. A negative residual (i.e., lower predicted than chronological age) reflects resistance; a positive residual indicates accelerated aging.^22^ For example, positive residuals derived from a model that regresses age on neuroimaging measures of gray matter (GM) volume indicate increased brain atrophy for a given age and thus accelerated brain aging.

Different measures of brain integrity served as predictors of chronological age in age-based residual methods (Table 1). Structural MRI measures were most commonly used, in the form of whole-brain voxel-wise maps of segmented GM^23-37^ or white matter (WM),^24,29-31,34,38^ regional cortical and subcortical volumes,^39-42^ regional cortical thickness or surface area,^42-45^ and raw images.^29,46,47^ Two studies additionally incorporated information on brain function like resting-state fMRI-derived functional connectome^40,43^ and regional homogeneity.^40^ Three others additionally used diffusion tensor imaging tract-based fractional anisotropy and diffusivity measures.^41,48,49^ Another study calculated age-based residuals from PET-derived measures of glucose metabolism, oxygen consumption, and blood flow,^50^ while another employed EEG-derived features of sleeping patterns.^e1^

Two studies derived age residuals from epigenetic data with senescence markers measured from postmortem tissue samples (dorsolateral prefrontal cortex DNA methylation levels^e2,e3^ and gene expression levels^e3^) used as predictors of age.

#### Brain-Based Residuals

In inverse models, chronological age can be used to predict brain status, as demonstrated by 2 studies in which data structures composed of imaging-derived measures of brain structure^48,e4^ and function^48^ were regressed onto chronological age.

### Residual Measures of Resilience

Similar to resistance-related residuals, several distinct yet comparable variations of resilience-related residuals were proposed in the literature, in the form of cognition-based or brain-based (Table 2 and eTable 6).

**Table 2:**
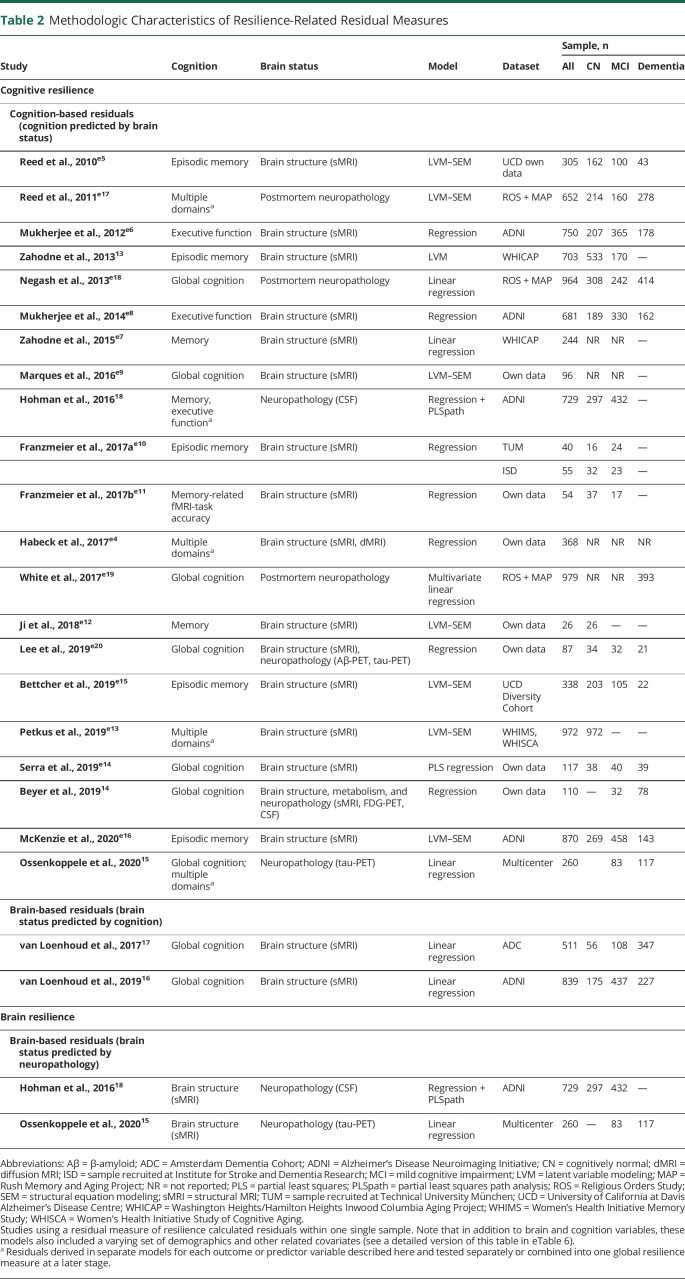
Methodologic Characteristics of Resilience-Related Residual Measures

#### Cognition-Based Residuals

Twenty-one studies operationalized cognitive resilience with a residual approach modeling cognition as outcome variable. The difference between observed and expected cognitive ability, as predicted by measures of brain structure or pathologic burden, yields an individual's deviation (from population norms) and captures resiliency or vulnerability to cerebral changes. Among the earliest proposed models is the residual memory variance model,^e5^ where cognitive resilience is conceptualized as residual variance in memory performance not explained by brain structure and demographics. Thirteen other studies derived comparable cognitive resilience measures from structural MRI data. The most common brain variables were volumes of whole-brain GM, hippocampus, and WM hyperintensities.^13,e4,e6-e16^ Three studies similarly regressed cognition onto postmortem neuropathologic measures.^e17-e19^ Two studies leveraged in vivo CSF biomarkers of AD pathology^15,18^ and 2 others explored a multimodal approach combining measures of brain structure and neuropathology to predict cognition.^14,e20^ The most commonly residualized cognitive abilities were (episodic or semantic) memory^13,15,18,e4,e5,e7,e10-e13,e15-e17^ and global cognition.^15,e9,e14,e18,e19^ Residuals of other cognitive domains were also explored^15,18,e4,e6,e8,e13,e17^ (eTable 5).

#### Brain-Based Residuals

Two studies proposed a brain-based residual measure of cognitive resilience where MRI-based whole-brain voxelwise GM maps^17^ or regional GM volumes^16^ were regressed onto global cognition scores.

Similarly, 2 other studies used brain-based residuals to quantify the degree to which structural integrity is preserved despite pathologic changes. Investigating brain resilience in the context of AD, these residual methods regressed MRI-based measures of brain structure, such as hippocampal volumes^18^ and cortical thickness,^15^ onto CSF biomarkers of β-amyloid^18^ and tau.^15,18^

### Analytical Approaches

Given that residuals quantify the difference between observed and predicted values of an outcome variable, a prediction model lies at the core of residual methods. Models built to capture normative patterns of brain and cognitive aging address a regression problem, that is, the prediction of continuous outcome measures such as cognition, age, and brain volumes. While the regression problem was virtually the same across studies, a wide range of analytical approaches was used to predict expected outcome values for the variables of interest, including data-driven and theory-driven approaches.

#### Analytical Models

Resistance-related residuals emerged from the notion of predicting chronological age from an individual's brain image, put forward by the machine learning community. Studies generally used a data-driven prediction-focused approach,^e21^ with models selected based on maximizing prediction accuracy in independent datasets. Consequently, increasingly sophisticated regression algorithms were employed to build accurate prediction models of the normative brain aging process. Models ranged from (regularized) multiple regression,^36-38,42,48,49,e1-e3^ nearest-neighbor,^e4^ linear support-vector^32,40,43-45^ and relevance-vector regression,^23-28,30^ Gaussian process regression,^31,35^ random forest,^43,50^ gradient boosting,^41^ and scaled subprofile modeling^39^ to most recently deep learning models such as convolutional neural networks.^29,34,46,47^ Residuals were generally calculated out-of-sample, with models trained on a training set and applied independently on the test individuals or within a cross-validation framework.

Residual measures capturing cognitive and brain resilience were developed within the field of psychometrics, hence the task was primarily addressed from a (more) theory-driven perspective. Rather than selecting models based on how well they predicted outcome variables of interest in test data, more causally plausible descriptive models that maximized model fit on the study sample were used. This is reflected in the statistical approaches used to model relationships between cognition, brain integrity, and neuropathology. The most prominent models were multivariable linear regression^14-17,e4,e6-e8,e10,e11,e18-e20^ and latent variable frameworks such as structural equation models^13,e5,e9,e12,e13,e15-e17^ and partial least squares regression.^18,e14^ Contrary to resistance residuals, resilience residuals were primarily derived in-sample. A priori defined measures of brain status (e.g., whole-brain atrophy) were more commonly used in resilience models, while resistance models relied more often on data-driven feature selection (e.g., principal component analysis of voxel-wise GM images).

#### Validation of Residual Measures

The brain age methods (measures of resistance) have undergone a more extensive methodologic validation, focusing on the model's predictive accuracy and generalizability,^23,25,28,29,31-37,40-43,45-47,50,e3^ test–retest^24,29,33,44,46^ and between-scanner reliabilities,^29^ longitudinal stability,^50^ and statistical robustness to different model measures.^23,34,36,49^ These measures were validated as biomarkers of brain health associated with cognitive decline,^24,25,27,44,e2,e3^ disease progression,^25,27,33^ and mortality.^31,e1^ Residual measures of cognitive and brain resilience were in turn more commonly tested for construct validity, that is, assessing whether residuals truly reflect the intended construct. This is challenging given the abstract conceptual nature of these constructs and absence of a gold standard. Researchers have predominantly used theoretical hypotheses derived from models of cognitive reserve^5^ to infer how a measure of cognitive resilience ought to operate. Tests of construct validity for cognitive resilience–related residuals assessed whether they correlated with established proxy variables (e.g., education, reading ability),^13,14,16,17,e4,e5,e17,e18,e20^ were associated with longitudinal cognitive decline^13-16,18,e5,e15,e16^ or with disease progression,^13,16-18,e5,e7,e13,e15^ and whether they moderated the effects of cerebral damage on cognition.^13,e5,e7,e14,e15,e20^

#### Role of Covariates

Models also differed in the selection of covariates. For example, some studies derived education-independent resilience measures,^13,14,e5-e9,e12-e16,e19^ measuring resilience beyond what is likely captured by education. Similarly, other studies estimated resilience while controlling for *APOE* genotype,^e11,e20^ thereby isolating resilience that is independent of this genetic component, which is known to contribute to people's resiliency or vulnerability to AD. In this way, a more environmentally driven resilience phenotype results from removing variance explained by certain genetic components, while a more genetically driven resilience measure results from removing influence of socioeconomic or lifestyle factors.

### Applications of Residuals

**Figure 4:**
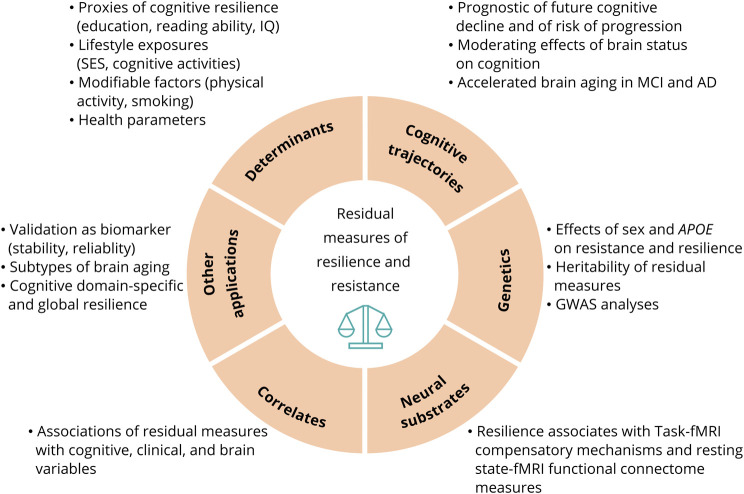
Applications of Residual-Method Based Measures of Resistance and Resilience This figure illustrates a summary of the diverse range of applications and analyses for which residual measures were employed. The studies included in the systematic review demonstrated the use of residual measures to facilitate research aimed to better understand the role of resistance and resilience in brain and cognitive aging. AD = Alzheimer disease; GWAS = genome-wide association study; MCI = mild cognitive impairment; SES = socioeconomic status.

The lack of effective pharmacologic therapies for neurodegenerative disorders demands increased scientific efforts focused on identifying modifiable environmental and lifestyle factors that enhance resistance and resilience (Figure 4). Residual measures capturing cognitive resilience were associated with education,^14-17,e4,e18,e20^ reading ability,^13,e4,e5,e18^ socioeconomic status,^e18^ occupation complexity,^e20^ and past leisure cognitive activity.^e17,e18^ Physical activity,^39^ education,^39,42^ and meditation^28^ were identified as protective factors associated with higher resistance, while various health factors^26,31,47,48^ (e.g., body mass index, blood pressure) and behaviors like smoking and alcohol consumption^42,48^ were identified as risk factors associated with lower resistance.

A promising application of residual measures is their use to identify genetic and functional mechanisms of resilience or resistance. Several studies have indicated that residuals partially capture heritable traits^29,34,e2,e6^ and can be utilized as primary phenotypes in genome-wide and pathway-based analyses^34,42,49,e6,e8,e19^ aiming to reveal genetic underpinnings of these phenomena. Furthermore, relating residuals to fMRI-based measures of brain's functional connectivity have revealed potential neural substrates of resilience and its underlying mechanistic processes (e.g., network efficiency and compensatory mechanisms).^30,e9-e12,e20^

Calculating residuals within a longitudinal framework allows investigation of the dynamic nature of resilience and resistance, as resilience capacity can be enhanced but also depleted over time. In this context, studies have shown that on average, levels of resilience and resistance decrease over time.^24,27,e7,e15^ Nevertheless, the rate of depletion might contain useful information in addition to baseline measures, as longitudinal change in residual measures of cognitive resilience was reported to predict dementia incidence^e7^ and cognitive decline.^e15^ Similarly, age-based resistance residuals changed more in individuals with progressive vs stable mild cognitive impairment (MCI).^24,27^

### Meta-analysis

Several studies investigated the association of residual measures with longitudinal cognitive decline and with risk of diagnostic progression. Of 54 studies, 10 were eligible for the meta-analysis^13-18,25,33,e5,e13^ (see excluded studies in eTable 4, available from Dryad, doi.org/10.5061/dryad.tx95x69xr), from which we extracted independent effect estimates for 3 separate meta-analyses.

#### Resistance and Risk of Progression

**Figure 5:**
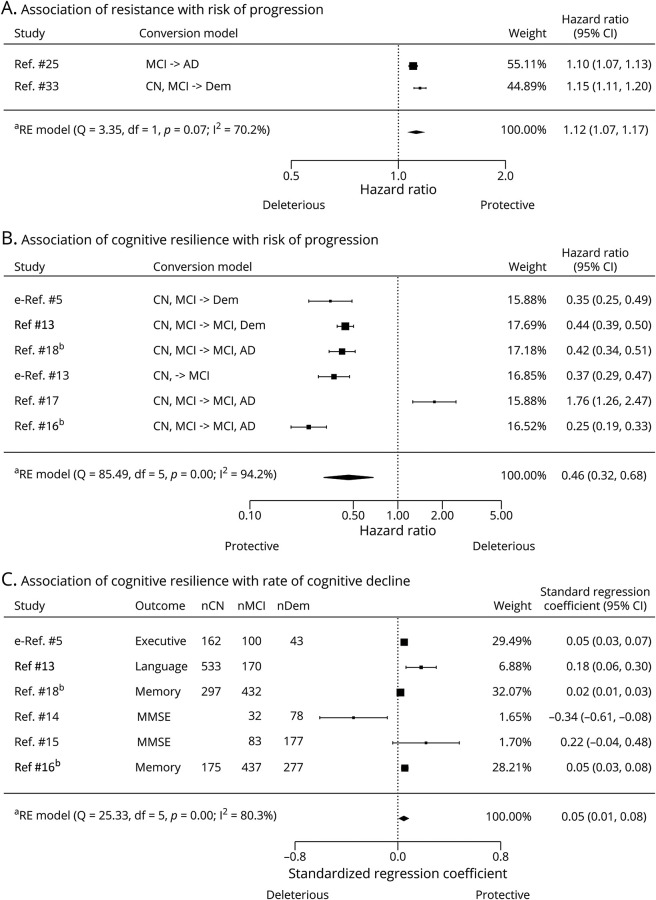
Forest Plots Illustrating Associations of Residual Method–Based Measures of Resistance and Resilience With Longitudinal Cognitive Outcomes (A) Random-effects meta-analysis of association between resistance and risk of progression to dementia/Alzheimer disease (AD). Note that in the age-based residual measures of resistance, a negative value indicates higher resistance level. Therefore, a hazard ratio >1 indicates a favorable outcome, such that a higher resistance level is associated with a reduced risk of converting to AD/dementia. (B) Random-effects meta-analysis of association between cognitive resilience and risk of progression to dementia/AD. In this case, the cognition-based or brain-based residual measures are proportional to the level of cognitive resilience, with a higher positive value indicating a higher cognitive resilience level. A hazard ratio <1 indicates a favorable outcome, where a higher cognitive resilience level is associated with a reduced risk of converting to AD/dementia. (C) Random-effects meta-analysis of association between cognitive resilience and rate of cognitive decline. A positive standardized regression coefficient indicates a favorable outcome, where a higher cognitive resilience level is associated with a slower rate of decline. The outcome indicates the specific cognitive domain/neuropsychological test used to assess cognitive decline in each study. Size of rectangles are proportional to the weight of the study in the random-effects model. CI = confidence interval; CN = cognitively normal; MCI = mild cognitive impairment; MMSE = Mini-Mental State Examination; nCN = number of cognitively normal patients; nDem = number of patients with dementia/AD; nMCI = number of patients with mild cognitive impairment; RE = random effects. ^a^RE model: random-effects model using the DerSimonian-Laird estimator. ^b^These 2 studies contain a partially overlapping sample of patients as they used the same cohort.

Two studies (n = 2,003) had suitable effect estimates for quantifying the association between baseline residual measures of resistance (based on structural MRI) and conversion to MCI or dementia^25,33^ (eTable 7, available from Dryad, doi.org/10.5061/dryad.tx95x69xr). The pooled effect of resistance residual measures (HR [95% confidence interval (CI)] 1.12 [1.07–1.17], *p* < 0.0001; Figure 5A) indicates that a lower level of resistance is associated with a higher risk of dementia. Moderate heterogeneity was detected (*I2* = 70.18%, *p* = 0.07).

#### Cognitive Resilience and Risk of Progression

Six studies (4 cognition-based, 2 brain-based, total n = 3,394) had suitable effect estimates for assessing associations between baseline residual measures of cognitive resilience and risk of disease progression^13,16-18,e5,e13^ (eTable 8, available from Dryad, doi.org/10.5061/dryad.tx95x69xr). The pooled analysis indicates an overall positive effect of cognitive resilience residual measures on risk of progression (HR [95% CI] 0.46 [0.32–0.68]; *p* < 0.001; Figure 5B). This suggests that across studies, a higher level of cognitive resilience is associated with reduced risk of MCI or dementia. High heterogeneity was detected (*I2* = 94.15%, *p* < 0.0001).

#### Cognitive Resilience and Rate of Cognitive Decline

Six studies (5 cognition-based, 1 brain-based, total n = 2,946) provided suitable effect estimates to assess associations between baseline residual measures of cognitive resilience and rate of cognitive decline.^13-16,18,e5^ Across studies, different cognitive and brain measures were used to calculate the residuals. Similarly, residuals were associated with decline in different cognitive outcomes while controlling for different covariates (eTable 9, available from Dryad, doi.org/10.5061/dryad.tx95x69xr). There was an overall positive effect of cognitive resilience residual measures on rate of cognitive decline (β[95% CI] 0.05 [0.01–0.08]; *p* < 0.01; Figure 5C). High heterogeneity was detected (*I2* = 80.26%, *p* = 0.0001).

As is evident from Figure 5, a few studies^14,17^ present conflicting results, possibly due to differences in disease severity across samples.^16^ Subgroup analyses or metaregression to further investigate this were precluded by the limited number of studies; nonetheless we provide stratified forest plots in eFigures 2 and 3, available from Dryad (doi.org/10.5061/dryad.tx95x69xr).

#### Risk of Bias and Publication Bias

Visual inspection of funnel plots indicated potential presence of publication bias (eFigure 1, available from Dryad, doi.org/10.5061/dryad.tx95x69xr). According to the domain-based assessment of bias, 3 studies^14,15,18^ showed moderate risk of bias (eTable 10, available from Dryad, doi.org/10.5061/dryad.tx95x69xr). Sensitivity analyses excluding these studies showed similar overall effects (eFigures 4 and 5, available from Dryad, doi.org/10.5061/dryad.tx95x69xr).

## Discussion

We reviewed and classified the residual measures proposed in the literature under the framework of resistance and resilience to brain and cognitive aging. We observed an increasing trend in employing residuals as operational measures of resistance and cognitive and brain resilience. There is considerable variability in how residuals are computed. Our meta-analyses show that despite large heterogeneity across studies, residual measures of resistance and cognitive resilience are associated with longitudinal cognitive trajectories. Higher resistance and resilience levels at baseline were associated with lower risk of clinical disease progression. Keeping the substantial between-study heterogeneity in mind, these results suggest that residual measures contain meaningful information as they explain individual variability in brain and aging trajectories.

The diverse implementations of residual methods and use of different samples across studies impair conclusions regarding superiority of one approach over others. The choice of method likely depends on the research question, available data, and expertise. For example, the brain and cognitive measures depend on what MRI sequences and neuropsychological tests are available. Similarly, model covariates must be selected with the research question in mind, that is, whether the researcher aims at isolating a more particular (e.g., genetically or environmentally driven) aspect of resilience that could potentially reveal a mechanistic process in more detail. Furthermore, the analytical implementation of these methods is expected to continue to change with the advent of further technological advancements (both in imaging technologies and in multivariate statistical models capable of handling increasing amounts of data).

### Critical Appraisal of Residual Methods

The operationalization of cognitive resilience with residual methods, initially proposed in the context of the overlapping framework of cognitive reserve,^e5^ has been scrutinized for its underlying methodology. With this approach, an error term is conceptualized as a quantitative measure of a theoretically significant concept. This appears somewhat problematic, especially given the lack of a gold standard that impedes a straightforward verification of construct validity. Although coined as (more) direct measures of resilience,^17,e5^ residuals are ultimately a parametrization of abstract concepts from observable variables, and ultimately embody the sum of what we are currently not able to measure and input directly in the model.^e15^ They were also referred to as “negative definitions” as they capture nonexplained variance, rather than apprehending what it does explain.^e4^ Residual measures, therefore, remain virtually agnostic of the underlying neural processes through which brain resilience, cognitive resilience, and resistance operate.^1^ A second critique of the residual approach is that its validity may depend on the variables included in the model.^e7,e22,e23^ For example, atrophy operationalized in different ways will result in different resilience scores for the same individual and there is currently no consensus about the optimal operationalization. Similarly, residuals also depend on how well all relevant pathologies are captured. In practice, often a resilience/resistance phenotype is measured (e.g., resilience to a specific pathology or in a specific cognitive domain), rather than overall capacities. A third methodologic challenge is that residuals also contain measurement error inherent to the imaging techniques and neuropsychological assessments,^3,e24^ and the extent to which this error (or the often encountered lack of accounting for it in the modeling framework) affects the residual measures is not clear. Taken together, this calls for a more comprehensive methodologic validation of residual measures; for example, one that assesses the implications of different variables and implementations on common well-characterized and representative datasets. Despite these limitations, the last decade has seen an increasing use of residual measures, as they present several advantages over established proxies. Residual methods are based on measures of brain integrity and pathologic burden, as well as cognition or chronological age, that is, elements that are fundamental to the theoretical definitions of resilience and resistance.^7^ Furthermore, they provide a straightforward and objective way to quantify between-individual variability with a person-specific score reflecting both its direction and its magnitude. This is useful because continuous-valued phenotypes are preferred over dichotomization of patients into resilient and nonresilient groups contingent on arbitrary thresholds.^e18^ When computed in a longitudinal framework, residuals enable measuring within-individual changes in resilience and resistance.^24,e7,e15^ This more accurately reflects the dynamic nature of these concepts, contrary to proxy variables, which often constitute static measures; for example, the maximum level of education attained. With incipient cerebral changes (e.g., aggregation of pathogenic proteins or atrophy), resilience manifests at the system-level in the form of an adaptive functional response; for example, hyperconnectivity.^e25^ During early stages of pathology, compensatory mechanisms^e26^ are triggered in resilient individuals, preserving cognitive function. However, as pathology progresses, resilience becomes depleted, resulting in accelerated decline. As shown by several studies,^14,16,e7,e15^ residual measures (essentially a function of cognition and pathology) can likely capture this dynamic “inverse U-curve” phenomenon. The findings summarized here provide cumulative evidence for residual methods as practical measures of resilience and resistance. Taken together, the results of the meta-analyses are consistent across studies and compatible with current theoretical models of brain and cognitive aging. This suggests that residual measures reflect resilience and resistance constructs and capture clinically meaningful information that explains future cognitive decline and disease progression.

With advancing technology, access to more accurate and detailed MRI-based measures of cerebral damage as well as PET-based measures of pathologic and physiologic processes is expected to increase. A more diverse and comprehensive set of pathology measures might explain a larger proportion of variance in clinical outcomes, resulting in smaller residuals. Several studies hypothesized that although residuals would decrease in magnitude, they would capture cognitive resilience more accurately.^13,e5,e17^ This hypothesis remains to be investigated. Because resilience and resistance arise from the complex interplay of genetics, environment, and experience, we are unlikely to be able to measure and model all the relevant variables (in a cost-effective way) in the near future. For that reason, the residual approach plays an important role in guiding current and future research into the aspects of genetics, biology, and environment that contribute to successful cognitive aging.

### The Road to Clinical Application

Research into determinants and mechanisms of successful cognitive aging, conveniently enabled by residual measures, has the potential to drive the development of early interventions to enhance resilience and resistance capacities. Due to their continuous and dynamic nature, residual measures could also be used as outcomes measured when assessing the effect of such interventions. Similarly, subject-level metrics of resilience could enable a more individualized prognosis of clinical outcomes (e.g., risk of cognitive decline/dementia).^18^ Converging evidence on the prognostic value of resistance and resilience residual measures suggests a potential role for these measures in patient management and clinical trial selection. Nonetheless, several questions remain to be investigated to establish feasibility, starting with a more rigorous methodologic validation. A comprehensive evaluation of different implementations of the residual measures is necessary to converge towards a clinically useful method. Furthermore, efforts should focus on developing practical measures that can be inferred from brain and cognitive data readily available in clinics. Other critical aspects are establishing accurate normative data representative of comprehensive reference groups and assessing their predictive value beyond other prognostic biomarkers. Further construct validation may be provided by observational studies replicating results of the studies reviewed here, or by use of residuals as outcomes in randomized controlled trials aimed at boosting resistance and resilience (e.g., lifestyle or pharmacologic interventions).

The implications of high scores on resilience and resistance scales in terms of predicting future disease progression need better understanding. Research to date suggested paradoxical effects, with cognitively resilient individuals showing slower decline in initial phases of disease^18,e5^ (relative to the less resilient), but more pronounced decline in later stages.^16,e15^ Finally, an important open research question is how resilience is differently manifested in normative aging compared to pathologic aging. Resilience is considered to represent a response to adverse changes, and is therefore expected to be measurable only when pathologic changes start or reach certain levels.^e16^ In our conceptual framework, we envisioned that resilience to normative aging-related processes also occurs and is appropriately quantifiable with residual methods that include a comprehensive set of senescent molecular, structural, or functional changes. However, the heterogeneity of samples used across the reviewed literature impedes determining the implications of residual measures in aging vs pathology. For example, many studies included community-dwelling elderly patients who are not fully characterized as being on a pathologic or nonpathologic aging trajectory. This question therefore needs further investigation, with some studies already taking steps in this direction.^e16,e20^

### Limitations

Our meta-analyses have several limitations. First, there were few studies available and the residuals of these studies likely capture distinct (yet overlapping) aspects of cognitive resilience (i.e., different phenotypes) as methods and variables varied. This warrants careful interpretation of the overall results. Second, the small number of studies precluded quantitative sensitivity analyses to explore sources of heterogeneity. Third, visual inspection of funnel plots indicated a potential risk of publication bias. Fourth, 2 studies contained overlapping samples^16,18^ and 3^14,15,18^ had moderate risk of bias (although their removal did not affect the overall effects greatly).

Our review and meta-analyses suggest that, despite methodologic differences, residual measures of resilience and resistance constitute useful measures that capture clinically meaningful information. Residuals facilitate, arguably to a larger extent than proxies, the ongoing research into determinants and mechanisms of resilience and resistance. Furthermore, they exhibit prognostic value in assessing risk of cognitive decline and progression to dementia that could ultimately be harnessed in clinical settings.

## Glossary

AD: Alzheimer disease
CI: confidence interval
GM: gray matter
HR: hazard ratio
MCI: mild cognitive impairment
WM: white matter
